# Efficacy of acupuncture in treating chemotherapy-related cognitive impairment in breast cancer patients: A systematic review and meta-analysis protocol

**DOI:** 10.1371/journal.pone.0318984

**Published:** 2025-02-06

**Authors:** Lishi Liu, Zihan Meng, Jun Yan

**Affiliations:** The Second Department of Oncology, The First Affiliated Hospital of Heilongjiang University of Chinese Medicine, Harbin, China; China Medical University, TAIWAN

## Abstract

**Background:**

Chemotherapy-related cognitive impairment (CRCI) is a common adverse effect of chemotherapy in breast cancer patients, with a high incidence that significantly impacts treatment adherence and quality of life. Currently, there is no definitive and effective treatment for CRCI. Research indicates that acupuncture may serve as a promising intervention for CRCI. This study aims to assess the effectiveness of acupuncture in treating CRCI.

**Methods:**

This study is guided by the Preferred Reporting Items for Systematic Reviews and Meta-Analysis Protocols (PRISMA-P). From the time of database construction to October 2024, we will search seven literature databases, three clinical study registry databases, and three sources of gray literature. The restricted languages are English and Chinese. We will use the Participants-Interventions-Comparators-Outcome-Conclusion-Study Design (PICOS) criteria to identify included studies and the RoB2 tool to assess the quality of the included studies. Data synthesis and meta-analysis will be performed using RevMan 5.3.

**Discussion:**

The results of this study will clarify the potential benefits of acupuncture in the treatment of CRCI and address existing disagreements about its efficacy. Furthermore, the findings will assist clinicians in making informed decisions regarding the treatment of CRCI.

**Trail registration:**

PROSPERO registration number: CRD42024568093.

## 1. Introduction

Breast cancer is the most common type of malignant tumor in women, and its incidence is increasing year by year, with a rising trend in younger patients [1]. According to the International Agency for Research on Cancer (IARC), there were 2.3 million new cases of breast cancer in 2022, with an incidence rate of 11.6%, making it the second most prevalent cancer globally [2]. In recent years, the use of various treatments such as surgery, chemotherapy, radiation, targeted therapy, and endocrine therapy has significantly reduced the risk of breast cancer recurrence [3–5], leading to an average 5-year survival rate of 91% among patients [1].

Chemotherapy, a frequently employed and critical treatment option, however, often causes multiple adverse effects. Beyond common side effects like nausea, vomiting, and fatigue, chemotherapy can also lead to chemotherapy-related cognitive impairment (CRCI) [6], colloquially termed "chemobrain" or "chemofog." A systematic review has demonstrated a strong correlation between chemotherapy and cognitive impairment [7].

The primary symptoms of CRCI include memory loss, difficulty concentrating, slower processing speed, and impaired executive functioning [8]. The incidence of CRCI varies widely, ranging from 17% to 70% [9–11], and can be as high as 75% in breast cancer patients [12]. Additionally, 35% of breast cancer patients experience persistent cognitive dysfunction even 3 to 4 years after chemotherapy [13, 14]. Although the severity of CRCI is generally milder than the cognitive impairments typically seen in patients with common neurological disorders [15], it still negatively affects treatment adherence, increases disease burden, and significantly impacts the quality of life for breast cancer patients, leading to considerable financial, emotional, and interpersonal costs [16–18].

The pathophysiology of CRCI is not well understood but can be broadly categorized into the direct neurotoxic effects and the indirect consequences of chemotherapeutic drugs. Chemotherapy can impair brain metabolism and cellular function through direct cytotoxic damage to neuronal cells. It may also induce CRCI via indirect mechanisms, such as treatment-induced cytokine disorders, oxidative stress, genetic polymorphisms, and hormone level changes triggered by treatment [19].

Current treatments for CRCI are limited and can be broadly categorized into pharmaceutical and nonpharmaceutical approaches [20, 21]. Pharmaceutical therapies include antidementia drugs (e.g., donepezil, memantine, and ginkgo biloba), central nervous system stimulants (e.g., methylphenidate and modafinil), and erythropoietin. However, the efficacy of these medications remains uncertain and may even cause undesirable side effects [22–24]. Therefore, large-scale studies are needed to clarify their therapeutic effects and mechanisms. Nonpharmacological interventions, such as cognitive training [25], psychological interventions [26], and exercise therapy [27], are now widely recommended, with pharmaceutical treatments typically considered only when other approaches fail [21]. A systematic review of 13 studies that evaluated three pharmaceutical interventions (CNS stimulants, erythropoietin alpha, and ginkgo biloba) and two nonpharmaceutical interventions (cognitive training and physical activity) found that pharmacologic treatments were ineffective, whereas cognitive training and physical activity improved CRCI [28].

Acupuncture is a crucial therapeutic tool in traditional Chinese medicine (TCM), whereby the insertion of needles into specific regions of the body (acupoints) and vibrations, electrical currents, or other forms of stimulation are administered to achieve therapeutic aims [29]. It is used to treat a variety of diseases and discomforts, including pain [30], insomnia [31], anxiety [32], and depression [33]. In a network meta-analysis of nonpharmacologic therapies for CRCI, acupuncture ranked third among all interventions; however, its inclusion in the literature was early, and only two studies were included [34]. In contrast, acupuncture did not achieve the anticipated impact in a systematic review. Multiple randomized controlled trials (RCTs) have demonstrated that acupuncture therapy enhances the management of CRCI in breast cancer patients [35–39], potentially attributed to an increase in brain-derived neurotrophic factor (BDNF) [39–42]. Further RCTs are presently being conducted to investigate the efficacy of acupuncture for treating CRCI in breast cancer [43]. Most RCTs have utilized the Montreal Cognitive Assessment (MoCA), the Mini-Mental State Examination (MMSE), and the Functional Assessment of Cancer Therapy-Cognitive Function (FACT-Cog) to evaluate CRCI [19, 20]. While these scales are widely recognized and reliable, they lack objectivity. Therefore, in our meta-analysis, we will utilize neuroimaging results and BDNF measures for subsequent analysis, as the data permits.

The objective of this study is to conduct a comprehensive systematic review and meta-analysis of the existing evidence regarding acupuncture as a treatment for CRCI. The aim is to evaluate the potential benefits of acupuncture in treating CRCI, address ongoing debates about its effectiveness, provide definitive information for clinical practice, and enhance the quality of life for patients with CRCI.

## 2. Methods

### 2.1 Study registration

This study protocol is registered with the PROSPERO International Prospective Register of Systematic Reviews (registration number CRD42024568093). The study will strictly abide by the guidelines of the Preferred Reporting Items for Systematic Reviews and Meta-Analysis Protocols (PRISMA-P). The checklist for the PRISMA 2015 statement can be found in Supporting Information S1 Checklist.

### 2.2 Inclusion criteria

The evaluation of study eligibility for inclusion will be based on the Participants-Interventions-Comparators-Outcome-Conclusion-Study Design (PICOS) criteria.

#### 2.2.1 Participants

Eligible participants must be female breast cancer patients aged 18 years or older who have undergone or are currently undergoing chemotherapy and who meet the criteria for cognitive impairment (MMSE score ≤ 23 or MoCA score ≤ 25) at the time of enrollment. There are no restrictions on the participants’ occupations or educational levels.

#### 2.2.2 Interventions

The primary intervention in the experimental group is acupuncture, without specified criteria for the number of acupuncture points, duration, or intensity. Participants in the experimental group may receive acupuncture alone or in combination with a control group.

#### 2.2.3 Comparators

The control group should receive conventional care or treatment, which includes sham acupuncture, pharmaceutical interventions (such as antidementia drugs, central nervous system stimulants, erythropoietin, and other herbal preparations), and nonpharmacological interventions (such as cognitive training, psychological interventions, and exercise therapy). The modalities of sham acupuncture include the following: 1. Sham acupuncture, in which needles are applied to areas that do not have a therapeutic effect; 2. Non-penetrating acupuncture, in which needles are applied using a needle set that does not penetrate the skin and resembles real needles; and 3. Superficial acupuncture, in which needles penetrate only the superficial layers of the skin and do not produce a therapeutic effect.

#### 2.2.4 Outcomes

Key outcomes include assessments of cognitive function and neuroimaging changes. The assessment scales are limited to the Montreal Cognitive Assessment (MoCA), the Mini-Mental State Examination (MMSE), the Functional Assessment of Cancer Therapy-Cognitive Function (FACT-Cog), the Auditory Verbal Learning Test (AVLT), the Trail Making Test (TMT), and the Controlled Oral Word Association Test (COWA) [19]. We will perform a neuroimaging assessment using magnetic resonance spectroscopy (MRS), focusing on changes in the metabolites N-acetylaspartate (NAA), choline (Cho), and myo-inositol (mI) [44]. Additionally, serum BDNF levels, which reflect the brain’s self-repair function, will be considered an important outcome [45].

#### 2.2.5 Study designs

All clinical RCTs relevant to the study topic will be included in this research, with language constraints limited to English and Chinese. Non-RCTs, observational studies, prospective studies, animal experiments, reviews, meta-analyses, and case reports will be excluded.

### 2.3 Exclusion criteria

1. patients with pre-existing neurological diseases should be excluded. 2. We will exclude non-RCTs, observational studies, prospective studies, animal experiments, reviews, meta-analyses, and case reports.

### 2.4 Search strategy

We will search seven literature databases, including PubMed, EMBASE, Web of Science, the Chinese Biomedical Literature Database, Cochrane Library, Wanfang Database, and the China National Knowledge Infrastructure (CNKI). Additionally, we will search for ongoing trials in three trial registry databases: ClinicalTrials, the EU Clinical Trials Register, and the WHO International Clinical Trials Registry Platform (ICTRP). Gray literature will be searched using Google Scholar, ProQuest, and ResearchGate. The time frame for the literature and data is from the construction of the repository to October 2024, with language limitations to English and Chinese. The search strategy for the PubMed database is shown in Table 1.

**Table 1:** Search strategies for PubMed databases.

No.	Search terms
#1	"Breast Neoplasms"[MeSH] OR "Breast cancer"[Title/Abstract] OR "Breast Neoplasms"[Title/Abstract] OR "Breast Tumors"[Title/Abstract] OR "Mammary Cancer"[Title/Abstract] OR "Breast Malignant Neoplasm"[Title/Abstract] OR "Breast Carcinomas"[Title/Abstract] OR "breast malignancies"[Title/Abstract]
#2	("Drug Therapy"[Mesh] OR "chemotherapy"[Title /Abstract] OR "chemical therapy"[Title/Abstract] OR "CHEMO"[Title/ Abstract]) AND ("Neurocognitive Disorders"[Mesh] OR "Cognition Disorders"[Mesh] OR "Cognitive Dysfunction"[Mesh])
#3	"Chemotherapy-Related Cognitive Impairment"[MeSH] OR "Chemotherapy Related Cognitive Impairment"[Title/Abstract] OR "CRCI"[Title/Abstract] OR "Chemotherapy-Related Cognitive Impairments"[Title/Abstract] OR "Chemotherapy-Related Cognitive Dysfunction"[Title/Abstract] OR "Chemotherapy Related Cognitive Dysfunction"[Title/Abstract] OR "Chemo-Fog"[Title/Abstract] OR "Chemo Fog"[Title/Abstract] OR "Chemobrain"[Title/Abstract]
#4	#2 OR #3
#5	"Acupuncture"[MeSH] OR "Acupuncture Therapy"[MeSH] OR "Acupuncture"[Title/Abstract] OR "Acupuncture Therapy"[Title/Abstract] OR "Acupuncture Treatment"[Title/Abstract] OR "Acupotomy"[Title/Abstract] OR "Electroacupuncture"[Title/Abstract]
#6	Randomized Controlled Trial[Publication Type] OR controlled clinical trial[Publication Type] OR randomized[Title/Abstract] OR randomly[Title/Abstract] OR trial[Title/Abstract] OR groups[Title/Abstract] OR placebo[Title/ Abstract]
#7	#1 AND #4 AND #5 AND #6

### 2.5 Study selection and data extraction

**Fig 1:**
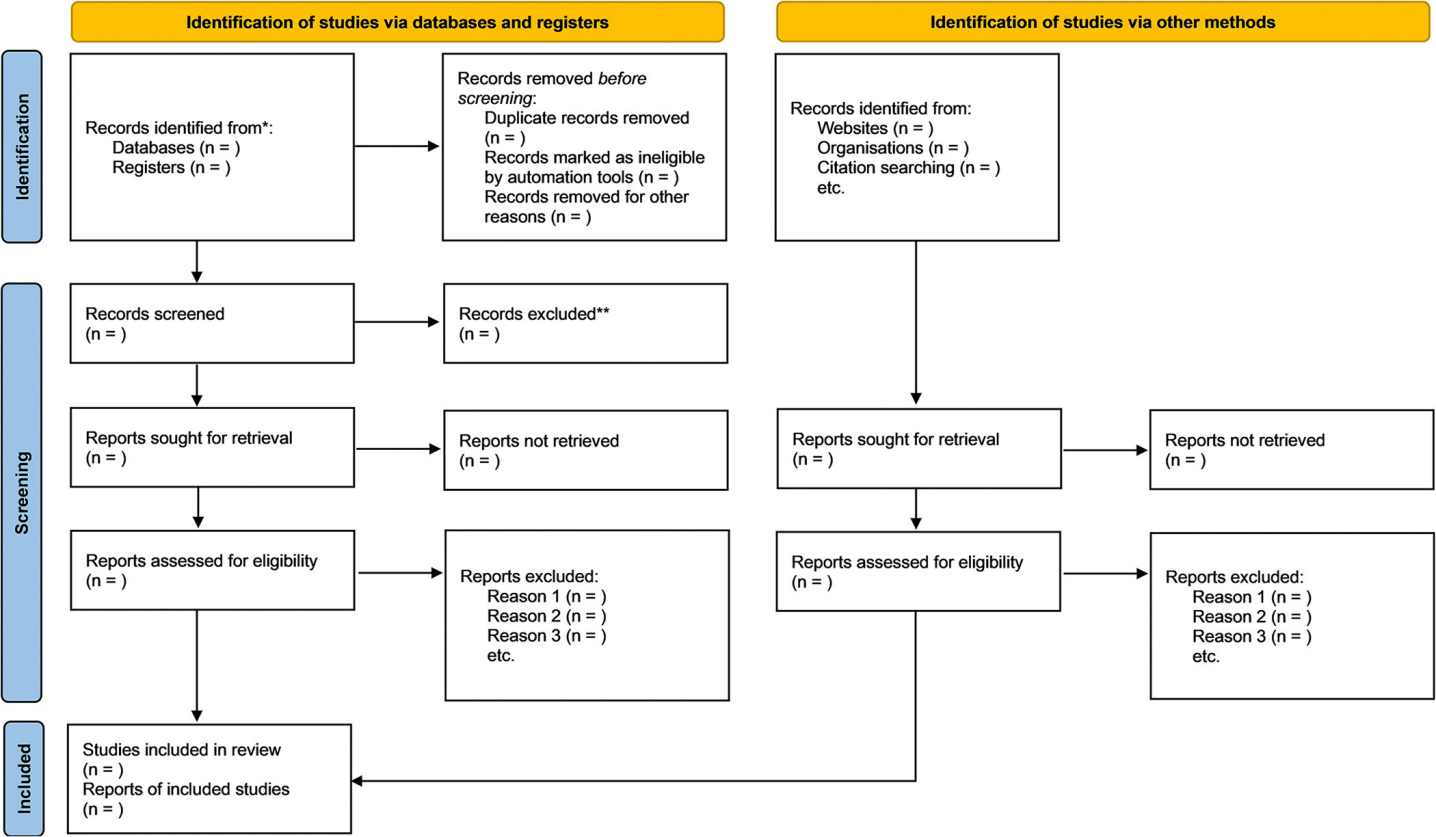
Flow chart of the study screening process.

Initially, we will import the collected material into Zotero software and remove duplicate studies. The screening process will be divided into two phases. In the first phase, two authors (LL and ZM) will independently examine the titles and abstracts of all studies to identify eligible ones based on the eligibility criteria. In the second phase, both authors will read the full texts of the eligible studies to verify their accuracy. Any conflicts that arise will first be discussed and resolved, with a third author (JY) consulted if necessary. Fig 1 illustrates the study screening process. The two authors (LL and ZM) will develop a numeric data extraction form to collect information from the included studies, including authors, year of publication, study design, sample size, participant characteristics, interventions and their details in both experimental and control groups, primary outcomes, and secondary outcomes. If any studies have incomplete or missing data, the corresponding authors will be contacted by email or phone for additional information.

### 2.6 Quality assessment

The quality of the included studies will be assessed using the Cochrane Risk of Bias Tool for Randomized Trials (RoB2). This tool comprises five primary domains: bias arising from the randomization process, deviations from intended interventions, missing outcome data, outcome measurement, and selective reporting. Each domain will be categorized as “low,” “high,” or “some concerns.” The quality assessment of all included studies will be conducted independently by two authors (LL and ZM). In the event of any differences, these will be resolved through open discussion among all authors.

### 2.7 Data synthesis and analysis

Data synthesis and analysis will be conducted using RevMan 5.4 software. For continuous outcomes, data will be analyzed using the mean difference (MD) and 95% confidence interval (CI) when the same or similar measures and units are used between studies, and using the standardized mean difference (SMD) and 95% CI when different measurement tools, scales, or units are employed. For dichotomous outcomes, data will be analyzed using the risk ratio (RR) and 95% CI when the event rate is high (>10%) and using the odds ratio (OR) and 95% CI when the event rate is low (<10%).

#### 2.7.1 Assessment of heterogeneity

Heterogeneity will be evaluated using the Q statistic and the I^2^ statistic and will be visually represented and analyzed using forest plots. With low heterogeneity (P ≥ 0.1, I^2^ ≤ 50%), the data will be analyzed using a fixed-effects model. If heterogeneity is high (P < 0.1, I^2^ > 50%), data will be analyzed using a random-effects model, with meta-regression and subgroup analysis to identify the sources of heterogeneity. Narrative analysis will be performed if heterogeneity is too great for meta-analysis.

#### 2.7.2 Subgroup analysis

To analyze sources of heterogeneity when it is high, or to explore correlations when it is low, we will conduct subgroup analyses when data permit. Criteria for subgroup grouping may include tumor metastasis, length of a single acupuncture session, type of acupuncture, and treatment course.

#### 2.7.3 Sensitivity analysis

We will perform sensitivity analyses using a case-by-case exclusion method to assess the robustness and reliability of the combined results.

#### 2.7.4 Assessment of the quality of evidence

The quality of evidence will be assessed using the Grading of Recommendations Assessment Development and Evaluation (GRADE) system. The GRADE quality assessment is categorized into five domains: risk of bias, inconsistency, indirectness, imprecision, and publication bias. The quality of evidence is classified into four grades: “high,” “moderate,” “low,” and “very low.” Two authors (LL and ZM) will independently evaluate the quality of evidence for each included study using GRADEpro GDT software, and any disputes will be resolved through discussion among all authors.

#### 2.7.5 Publication bias

When more than ten studies are included, publication bias will be analyzed using the Egger test and funnel plots. When fewer than ten studies are included, publication bias will be analyzed using the Begg test and funnel plot.

### 2.8 Ethics and dissemination

This study is a systematic review and meta-analysis using published data and does not require ethical approval.

## 3. Discussion

Breast cancer patients treated with chemotherapy have a high likelihood of developing CRCI. Although this cognitive impairment is typically modest to moderate, it nonetheless significantly affects patients’ treatment processes and quality of life. The efficacy of pharmaceutical therapies for CRCI is not particularly clear; they do not demonstrate advantages over nonpharmacological therapies and can have negative side effects. Therefore, nonpharmacological therapies are preferable for treating CRCI. The effectiveness of acupuncture, a traditional Chinese medicine therapy, in treating cognitive impairment is recognized in clinical practice, but its efficacy for CRCI remains debated. While prior systematic reviews and network meta-analyses have assessed acupuncture’s effectiveness for CRCI, their findings are limited due to the small number of included studies, older publication dates, and reliance on subjective outcomes. More recent studies on acupuncture for CRCI have utilized rigorous research methods, more objective scales, and outcomes such as neuroimaging results and BDNF measurements. This systematic review will analyze and synthesize these studies to provide reliable results that will enhance the treatment of patients with CRCI and enable clinicians to make clearer therapeutic decisions.

## Supporting information

S1 Checklist PRISMA 2015 checklist. (DOCX)
